# The Extended N-Terminal Domain Confers Atypical Chemokine Receptor Properties to CXCR3-B

**DOI:** 10.3389/fimmu.2022.868579

**Published:** 2022-06-01

**Authors:** Giulia D’Uonnolo, Nathan Reynders, Max Meyrath, Dayana Abboud, Tomasz Uchański, Toon Laeremans, Brian F. Volkman, Bassam Janji, Julien Hanson, Martyna Szpakowska, Andy Chevigné

**Affiliations:** ^1^ Immuno-Pharmacology and Interactomics, Department of Infection and Immunity, Luxembourg Institute of Health, Esch-sur-Alzette, Luxembourg; ^2^ Faculty of Science, Technology and Medicine, University of Luxembourg, Esch-sur-Alzette, Luxembourg; ^3^ Laboratory of Molecular Pharmacology, GIGA-Molecular Biology of Diseases, University of Liège, Liège, Belgium; ^4^ Confo Therapeutics, Ghent, Belgium; ^5^ Department of Biochemistry, Medical College of Wisconsin, Milwaukee, WI, United States; ^6^ Tumor Immunotherapy and Microenvironment, Department of Oncology, Luxembourg Institute of Health, Luxembourg City, Luxembourg; ^7^ Laboratory of Medicinal Chemistry, Centre for Interdisciplinary Research on Medicines (CIRM), University of Liège, Liège, Belgium

**Keywords:** ACKR3, ACKR2, CXCR3B, CXCL11/I-TAC, CXCL10/IP-10, arrestin, scavenger, isoform

## Abstract

The chemokine receptor CXCR3 plays a critical role in immune cell recruitment and activation. CXCR3 exists as two main isoforms, CXCR3-A and CXCR3-B, resulting from alternative splicing. Although the two isoforms differ only by the presence of an N-terminal extension in CXCR3-B, they have been attributed divergent functional effects on cell migration and proliferation. CXCR3-B is the more enigmatic isoform and the mechanisms underlying its function and signaling remain elusive. We therefore undertook an in-depth cellular and molecular comparative study of CXCR3-A and CXCR3-B, investigating their activation at different levels of the signaling cascades, including G protein coupling, β-arrestin recruitment and modulation of secondary messengers as well as their downstream gene response elements. We also compared the subcellular localization of the two isoforms and their trafficking under resting and stimulated conditions along with their ability to internalize CXCR3-related chemokines. Here, we show that the N-terminal extension of CXCR3-B drastically affects receptor features, modifying its cellular localization and preventing G protein coupling, while preserving β-arrestin recruitment and chemokine uptake capacities. Moreover, we demonstrate that gradual truncation of the N terminus leads to progressive recovery of surface expression and G protein coupling. Our study clarifies the molecular basis underlying the divergent effects of CXCR3 isoforms, and emphasizes the β-arrestin-bias and the atypical nature of CXCR3-B.

## 1 Introduction

Chemokine receptors are class A seven transmembrane domain G protein-coupled receptors (GPCRs) that bind small, structurally conserved cytokines with chemotactic properties, referred to as chemokines. Chemokine receptors are classified into four subfamilies (CCR, CXCR, XCR, and CX3CR) according to distinct cysteine motifs within the N terminus of the chemokines that they recognize. Chemokines and their receptors form an intricate network in which a chemokine can usually bind to many receptors, and a receptor recognizes several chemokines (1, 2).

Over the last decades, a new subfamily of chemokine receptors, referred to as ‘atypical’ chemokine receptors (ACKRs) and presently comprising four members (ACKR1–4), has emerged as important regulators of the chemokine network (2, 3). ACKRs differ from the ‘classical’ chemokine receptors notably by their inability to elicit G protein-mediated signaling, while most of them conserved the ability to recruit β-arrestin in response to chemokines. The molecular basis of the lack of G protein coupling remains elusive but has been partly attributed to alterations in the DRY motif and structural particularities in their intracellular pocket that preclude efficient G protein binding or activation (2, 4). Despite the absence of G protein signaling upon activation, ACKRs modulate cell migration and physiological processes by regulating the availability of chemokines for the classical receptors, among others through their internalization. This activity was previously considered to mainly rely on β-arrestins (4–8) but recent studies showed that alternative mechanisms may also drive the regulatory functions of ACKRs (9–12). Other distinctive properties of ACKRs are their unconventional cellular localization, trafficking and expression profile. ACKRs are indeed predominantly found in endosomal vesicles and are generally expressed on endothelial cells and epithelial cells of barrier organs as opposed to classical chemokine receptors that are mostly present at the cell surface of hematopoietic and immune cells (13, 14).

The chemokine receptor CXCR3 is mainly present on activated immune cells and mediates their migration towards sites of inflammation but it is also expressed on barrier cells as well as on cancer cells and within the tumor microenvironment (15–17). In humans, the gene encoding for CXCR3 can be transcribed to three alternative splice variants: CXCR3-A, CXCR3-B, and CXCR3-Alt, which give rise to structurally distinct proteins. CXCR3-B bears an extended N terminus wherein a 51-amino-acid stretch replaces the first four residues of CXCR3-A, while CXCR3-Alt lacks two transmembrane regions and shows a modified C terminus (**Supplementary Figure 1**) (18, 19). The three isoforms also exert different cellular functions in response to their chemokine ligands CXCL11, CXCL10, and CXCL9. The biology of CXCR3-Alt is not well investigated and although receptor internalization upon cognate chemokine binding has been described, no G_i_ protein activation nor β-arrestin recruitment could be observed (20). More efforts have been put in investigating the two other isoforms, which have been attributed opposing cellular functions. Indeed, CXCR3-A, mainly present on leukocytes, mediates cell migration and proliferation through activation of G_i_ and calcium signaling (20–26). In contrast, CXCR3-B is reported to be abundantly expressed on barrier cells (27), to inhibit cell migration and proliferation, and to induce apoptosis upon ligand stimulation (18, 28–30). However, although a β-arrestin bias was proposed for CXCR3-B (20, 21), the molecular mechanism underlying the receptor functions remain unclear.

The divergent cellular effects and expression patterns of the two CXCR3 isoforms led us to hypothesize that CXCR3-B could act as an ACKR. Thus, we investigated the profile of CXCR3-B with regard to the established features of ACKRs, namely the absence of G protein coupling, the predominant intracellular location and the scavenging properties. Using a large panel of live cell-based assays to monitor G protein and β-arrestin transducers, we showed that CXCR3-B does not signal *via* G proteins, while it maintains its ability to recruit β-arrestins. Furthermore, we observed important intracellular pools of CXCR3-B, which could be mobilized upon chemokine stimulation. Finally, we demonstrated herein that the N-terminal extension of CXCR3-B considerably alters its subcellular distribution and signaling capacity without changing the binding mode and selectivity of its chemokine ligands. Hence, we propose that the two CXCR3 isoforms could be regarded as distinct effectors in analogy to classical and atypical chemokine receptors.

## 2 Material and Methods

### 2.1Chemokines and Antibodies

#### 2.1.1 Native Chemokines

CXCL11, CXCL10, CXCL9, CXCL12, and CCL5 were purchased from Peprotech.

#### 2.1.2 Fluorescently Labeled Chemokines

CXCL11, CXCL10, CXCL9, and CCL5 coupled to Alexa Fluor 647 were purchased from Protein Foundry, LLC.

#### 2.1.3 Custom CXCL11 Chemokines

The N-terminally truncated, P2G-mutated and N-loop chimeras were produced as previously described (2, 31–33). In brief, cells were grown in Terrific Broth and production of modified CXCL11, cloned into pQE30 vectors, was induced with 1 mM isopropyl β-D-1-thiogalactopyranoside. Cell pellets were then lysed, centrifuged at 12 000 x g for 20 minutes and the supernatant and solubilized inclusion body pellets were added to nitrilotriacetic acid resin for 1 hour. Bound proteins were eluted with 6 M guanidinium chloride, 50 mM Na_2_PO_4_ (pH 7.4), 300 mM NaCl, 500 mM imidazole, 0.2% sodium azide and 0.1% β-mercaptoethanol, the eluate pooled and refolded *via* dilution overnight before cleavage of the His6SUMO fusion tag by Ulp1 protease for 4 hours. The His6SUMO fusion tag and chemokine were separated using cation-exchange and the eluate subjected to reverse-phase high-performance liquid chromatography as a final purification.

#### 2.1.4 Chemokine Processed by Dipeptidyl Peptidase 4

CXCL11, CXCL10, CXCL9 and CCL5 (9 µM) were incubated with recombinant dipeptidyl peptidase 4 (CD26) (100 U) in 20 µl Tris/HCl 50 mM pH7.5 + 1 mM EDTA for 90 minutes at 37°C.

#### 2.1.5 Antibodies

Phycoerythrin-conjugated anti-CXCR3 (1C6) and anti-CXCR4 (12G5) mAbs were purchased from BD Biosciences. Allophycocyanin-conjugated anti-ACKR2 (196124) was from R&D Systems and anti-ACKR3 (8F11-M16) from BioLegend.

### 2.2 Cell Culture

HEK293T and U-87 MG cells were grown in Dulbecco’s modified Eagle medium (DMEM) supplemented with fetal bovine serum (10 and 15% respectively) and penicillin/streptomycin (100 units/mL and 100 µg/mL). HEK293T.CXCR3-A or HEK293T.CXCR3-B cell lines stably expressing CXCR3 isoforms were established using pIRES-hygromycin vector and antibiotic selection and were grown in DMEM medium supplemented with 100 µg/mL hygromycin. Stable cell lines HEK293T-ACKR3 (34) and HEK293T-ACKR2 (35) were grown in DMEM medium supplemented with 5 µg/mL puromycin and 200 µg/mL hygromycin, respectively. HEK293T.pGlo, stably expressing cAMP GloSensor (GloSensor-22F cAMP, Promega) were grown in DMEM medium supplemented with 150 µg/mL hygromycin.

### 2.3 Recruitment Assays

#### 2.3.1 NanoBiT assay

miniG protein (mG, engineered GTPase domain of G_α_ subunit), β-arrestin-1 and β-arrestin-2 recruitment to WT and modified receptors (CXCR3-A, CXCR3-B, CXCR3-B N-terminally truncated isoforms, sp-IL6-CXCR3-B, where CXCR3-B sequence was preceded by Interleukin-6-derived signal peptide MNSFSTSAFGPVAFSLGLLLVLPAAFPAP, CXCR4, and extCXCR4), was monitored using a nanoluciferase complementation-based assay (NanoBiT, Promega) (36–38). 4 x 10^6^ HEK293T cells or 1.5 x 10^6^ U-87 MG cells were plated in 10-cm dishes and cultured for 24 or 48 hours, respectively, before transfection with vectors encoding for miniG proteins or human β-arrestins N-terminally fused with LgBiT and the chemokine receptors C-terminally fused with SmBiT. 48 hours after transfection, cells were harvested, incubated for 20 minutes at 37°C with coelenterazine H in Opti-MEM, and distributed into white 96-well plates (1.5 x 10^5^ cells/well). Chemokine ligands were then added and the luminescence generated upon nanoluciferase complementation was measured with a Mithras LB940 luminometer (Berthold Technologies).

#### 2.3.2 NanoBRET Assay

β-arrestin-1 and β-arrestin-2 recruitment to CXCR3-A and CXCR3-B upon chemokine-stimulation was monitored using NanoBRET (Promega) (35, 39). 4 x 10^6^ HEK293T cells were plated in 10-cm dishes and cultured for 24 hours before transfection with vectors encoding for the human β-arrestin-1 or β-arrestin-2 N-terminally fused with nanoluciferase and the chemokine receptor isoforms C-terminally fused with mNeonGreen. 48 hours after transfection, cells were harvested, incubated with coelenterazine H in Opti-MEM and immediately distributed into black 96-well plates (1.5 x 10^5^ cells/well). Upon addition of chemokines at the indicated concentrations, BRET signal was measured with a GloMax plate reader (Promega) equipped with 450/10 filter for donor luminescence emission and 530 LP filter for acceptor fluorescence emission. BRET signal is defined as acceptor/donor ratio. Ligand-induced changes in BRET ratio were expressed as ΔBRET, which was calculated as follows: (Ratio_stimulated_− Ratio_basal_)/Ratio_basal_] × 100.

### 2.4 G Protein Dissociation

Ligand-mediated G protein dissociation was monitored using BRET-based G protein activity sensors (40). These biosensors are based on a tricistronic plasmid which encodes nanoluciferase-tagged G_α_ subunits together with the related G_β_ and circularly permutated Venus tagged G_γ_. G protein activity is monitored through the reduction of BRET signal upon G protein subunit dissociation. 4 x 10^6^ HEK293T cells were plated in 10-cm dishes, cultured for 24 hours before transfection with BRET sensors and untagged CXCR3-A or CXCR3-B. 48 hours after transfection, cells were harvested, incubated with coelenterazine H in Opti-MEM and immediately distributed into black 96-well plates (10^5^ cells/well). Upon addition of chemokines at the indicated concentrations, BRET signal was measured with a GloMax plate reader (Promega) equipped with 450/10 filter for donor luminescence emission and 530 LP filter for acceptor fluorescence emission. BRET signal is defined as acceptor/donor ratio. Ligand-induced changes in BRET ratio were expressed as ΔBRET, which was calculated as follows: (Ratio_stim_− Ratio_basal_)/Ratio_basal_] × 100.

### 2.5 cAMP Measurements

cAMP measurements upon chemokine stimulation were performed using a luminescence GloSensor cAMP reporter assay (Promega) (41). 4 x 10^6^ HEK293T.pGlo cells were plated in 10-cm dishes and cultured for 24 hours before transfection with CXCR3-A or CXCR3-B-encoding pIRES vectors or empty vector. 48 hours later, cells were harvested, incubated for 1 hour at 37°C with the firefly luciferase substrate and IBMX (300 µM) in HBSS (120 mM NaCl, 5.4 mM KCl, 0.8 mM MgSO_4_, 10 mM HEPES, pH 7.4, 10 mM glucose) and distributed into white 96-well plates (1 x 10^5^ cells per well). cAMP-dependent changes in luminescence in response to chemokines at the indicated concentrations was measured with a Mithras LB940 luminometer (Berthold Technologies).

### 2.6 Intracellular Calcium Mobilization

CXCR3-driven chemokine-induced calcium flux was assessed using an assay based on nanoluciferase complementation (NanoBiT) and Ca^2+^-dependent calmodulin–MYLK2S protein association (42). HEK293T cells were plated in a 6-well plate (1 x 10^6^ cells per well) and cultured for 24 hours before transfection with CXCR3-A- or CXCR3-B-encoding pIRES vectors and plasmids encoding for calmodulin C-terminally fused to SmBiT and MYLK2S N-terminally fused to LgBiT. 48 hours after transfection, cells were incubated in PBS supplemented with 1 mM CaCl_2_ and 0.5 mM MgCl_2_ for 10 minutes at 37°C. Coelenterazine H was then added, cells distributed in a 96-well plate (10^5^ cells per well) and incubated for 20 minutes at 37°C. The baseline signal was acquired for 2 minutes. Calcium flux upon stimulation with chemokines (100 nM) or the calcium ionophore A23187 (1 µM) were quantified using the changes in luminescence measured on a GloMax plate reader (Promega).

Indo-1 AM ratiometric fluorescent indicator was also used as additional readout. HEK293T, HEK293T cells stably expressing CXCR3-A or CXCR3-B were incubated with 1 µM of Indo-1 AM (Thermo Fisher Scientific) in PBS supplemented with 1 mM CaCl_2_ and 0.5 mM MgCl_2_ for 1 hour at 37°C. Cells were pelleted, resuspended in PBS/CaCl_2_/MgCl_2_, distributed in a 96-well plate (10^5^ cells per well) and incubated for 30 minutes. First the baseline signal was acquired for 2 minutes. Cells were then stimulated with chemokines (100 nM) and the calcium flux was measured for 2 minutes. The validity of the assay was confirmed using the calcium ionophore A23187 (1 µM). Fluorescence was acquired on a GloMax plate reader using the 365 nm excitation laser and 415–445 and 495–505 emission filters to evaluate calcium-bound and free Indo-1, respectively.

### 2.7 Transcriptional Nanoluciferase Reporter Assays

Activation of the MAPK/ERK-, RhoA-, prolonged calcium- and cAMP-dependent signaling pathways was evaluated using a serum response element (SRE), Serum Response Factor Response Element (SRF-RE), Nuclear Factor of Activated T-cell Response Element (NFAT-RE) and a cAMP response element (CRE) nanoluciferase reporter assay, respectively (Promega). 1 x 10^6^ HEK293T cells were seeded in a 6-well plate, cultured for 24 hours, and co-transfected with the pNL3.2.SRE, pNL3.2.NFAT-RE, pNL3.2.SRF-RE or pNL3.2.CRE, and CXCR3-A- or CXCR3-B-encoding pIRES vectors. 24 hours after transfection, 5 x 10^4^ cells/well were seeded in a white clear-bottom 96-well plate and 24 hours later the medium was replaced by serum-free DMEM and incubated for 30 minutes at 37°C. Chemokines (100 nM) and corresponding positive control (50 nM phorbol 12-myristate 13-acetate (PMA) for SRE, 10% FBS for CRE, 1 µM ionomycin for NFAT, 10% FBS for SRF) were then added and incubated for 6 hours. Coelenterazine H was then added and luminescence was read over 20 minutes on a Mithras LB940 plate reader (Berthold Technologies).

### 2.8 Receptor Cellular Distribution Assays

#### 2.8.1 Fluorescent Microscopy

3 x 10^4^ HEK293T cells transiently transfected with CXCR3-A, CXCR3-B, ACKR3, or ACKR2 C-terminally fused to mNeonGreen were seeded in a poly-lysine-coated µ-Slide 8-well-chambered coverslip (Ibidi). After 24 hours, cells were washed twice with PBS and fixed with 3.5% (w/v) paraformaldehyde solution for 20 minutes at RT. Cells were washed three times with PBS and incubated with anti-CXCR3 (1C6), anti-ACKR3 (8F11-M16), and anti-ACKR2 (196124) mAb for one hour at 4°C. Cells were then washed twice with PBS and incubated for 20 minutes at RT with Hoechst 33342 dye (1 µg/mL). Cells were washed twice with PBS before acquiring images on a Zeiss LSM880 confocal microscope using a 63x oil-immersion objective and Zen Black 2.3 SP1 software (Zeiss).

#### 2.8.2 Flow Cytometry

To determine chemokine receptor subcellular distribution, HEK293T cells transiently transfected with plasmids encoding CXCR3-A or CXCR3-B C-terminally fused with mNeonGreén or with an empty vector were used. 48 hours after transfection, 1.5 x 10^5^ cells were seeded in a 96-well plate and incubated with anti-CXCR3 (1C6) mAb or isotype control for 45 minutes at 4°C, washed twice with FACS buffer (PBS, 0.1% sodium azide, 1% BSA) and then incubated for 20 minutes at 4°C with Zombie NIR viability dye, before measuring the fluorescence on a Quanteon Flow Cytometer (NovoCyte).

To monitor receptor cycling, 1.5 x 10^5^ HEK293T.CXCR3-A, HEK293T.CXCR3-B, HEK293T.ACKR3 (34) or HEK293T.ACKR2 (35) cells were seeded in a 96-well plate and incubated for 3 hours with 0.1 mg/mL proteinase K to remove extracellular epitopes. Cells were washed twice with PBS and incubated for one additional hour at 37°C in DMEM supplemented with 50 µM cycloheximide to measure re-surfacing receptors. Cells were then washed twice with PBS and an excess of anti-CXCR3 (1C6), anti-ACKR3 (8F11-M16), or anti-ACKR2 (196124) mAb was added and incubated for 45 minutes at 4°C. Cells were then washed once with PBS and incubated for 20 minutes at 4°C with Zombie Green viability dye (BioLegend). After two PBS washes surface receptor expression was measured on a Quanteon Flow Cytometer (NovoCyte).

To follow receptor subcellular distribution after chemokine stimulation, 1.5 x 10^5^ HEK293T.CXCR3-A, HEK293T.CXCR3-B, HEK293T.ACKR3 or HEK293T.ACKR2 cells were seeded in a 96-well plate, incubated or not with the V ATPase inhibitor, bafilomycin A1 (1.5 µM), for 40 minutes and then stimulated with chemokines (100 nM) for 10, 20, 40 or 60 minutes at 37°C in medium containing 50 µM cycloheximide. Cells were then washed twice with PBS and incubated for 40 minutes at 37°C, in the absence of chemokines to allow receptors re-surfacing. Cells were washed once with a low-pH buffer (50 mM glycine, 150 mM NaCl, pH 3.5) and twice with PBS. An excess of receptor-specific antibody was then added and incubated for 45 minutes at 4°C. Cells were then washed once with PBS and incubated for 20 minutes at 4°C with Zombie Green viability dye (BioLegend). The receptor surface expression was measured on a Quanteon Flow Cytometer (NovoCyte).

#### 2.8.3 Surface Nanoluciferase Complementation (HiBiT)

Receptor cellular distribution, in basal conditions and upon ligand stimulation, was monitored by nanoluciferase complementation assay. In brief, chemokine-induced changes in surface receptor levels were monitored with the use of Nano-Glo HiBiT extracellular detection system (Promega). 4 x 10^6^ HEK293T cells were plated in 10-cm dishes and cultured for 24 hours before transfection with pHiBiT vectors encoding for CXCR3 isoforms N-terminally fused to HiBiT. 48 hours later, cells were distributed in white 96-well plates (5 x 10^4^ cells per well) and stimulated with chemokines (100 nM) for 5, 10, 20, 40 minutes at 37°C. After addition of soluble LgBiT protein, luminescence was recorded over 30 minutes with a GloMax plate reader (Promega). In unstimulated conditions, surface and total receptor expression was determined using Nano-Glo HiBiT extracellular detection system (Promega) and Nano-Glo HiBiT lytic detection system (Promega), respectively.

### 2.9 Chemokine Uptake and Binding

#### 2.9.1 Flow Cytometry

To monitor chemokine uptake and binding, 1.5 x 10^5^ HEK293T cells transiently transfected with vectors encoding CXCR3-A or CXCR3-B C-terminally fused to mNeonGreen were incubated for 1 hour at 37°C or 4°C in the presence of 33 nM Alexa Fluor 647-labeled chemokines (Protein Foundry). Cells were washed twice with FACS buffer and afterwards subjected or not to proteinase K treatment (0.1 mg/mL) for 3 hours at 4°C to evaluate and compare unspecific chemokine binding to the cell surface. Cells were washed twice with FACS buffer and then incubated for 20 minutes at 4°C with Zombie NIR viability dye (BioLegend). After two PBS washes, the fluorescent chemokine uptake was quantified using a Quanteon Flow Cytometer (NovoCyte).

#### 2.9.2 Confocal Microscopy

3 x 10^4^ HEK293T cells transiently transfected with vectors encoding CXCR3-A or CXCR3-B C-terminally fused to mNeonGreen were seeded in a poly-lysine-coated µ-Slide 8-well-chambered coverslip (Ibidi) and grown for 24 hours. Cells were incubated for 1 hour at 37°C with 100 nM of Alexa Fluor 647-labeled chemokines (Protein Foundry) and co-incubated one additional hour with 750 nM LysoTracker™ Red DND-99 (ThermoFisher). Cells were then washed twice with PBS, fixed with 3.5% (w/v) paraformaldehyde for 20 minutes at RT, and washed twice with PBS. Nuclear staining was performed with Hoechst 33342 dye (1 µg/mL) for 20 minutes at RT and cells were washed three times with PBS. Images were acquired with a Zeiss LSM880 confocal microscope using a 63x oil-immersion objective and Zen Black 2.3 SP1 software (Zeiss).

### 2.10 Data and Statistical Analysis

Concentration-response curves were fitted to the three-parameter Hill equation using an iterative, least-squares method (GraphPad Prism version 9.3.0) to provide EC_50_, % maximum values and standard errors of the mean. All curves were fitted to data points generated from the mean of at least three independent experiments. All statistical tests i.e. ordinary or repeated measures one way- and two-way ANOVA, unpaired t-tests, Kruskal-Wallis and Mann-Whitney tests were performed with GraphPad Prism 9.3.0.

## 3 Results

### 3.1 The CXCR3-B Isoform Is Not Coupled to G Proteins but Maintains β-Arrestin Recruitment Capacity

**Figure 1:**
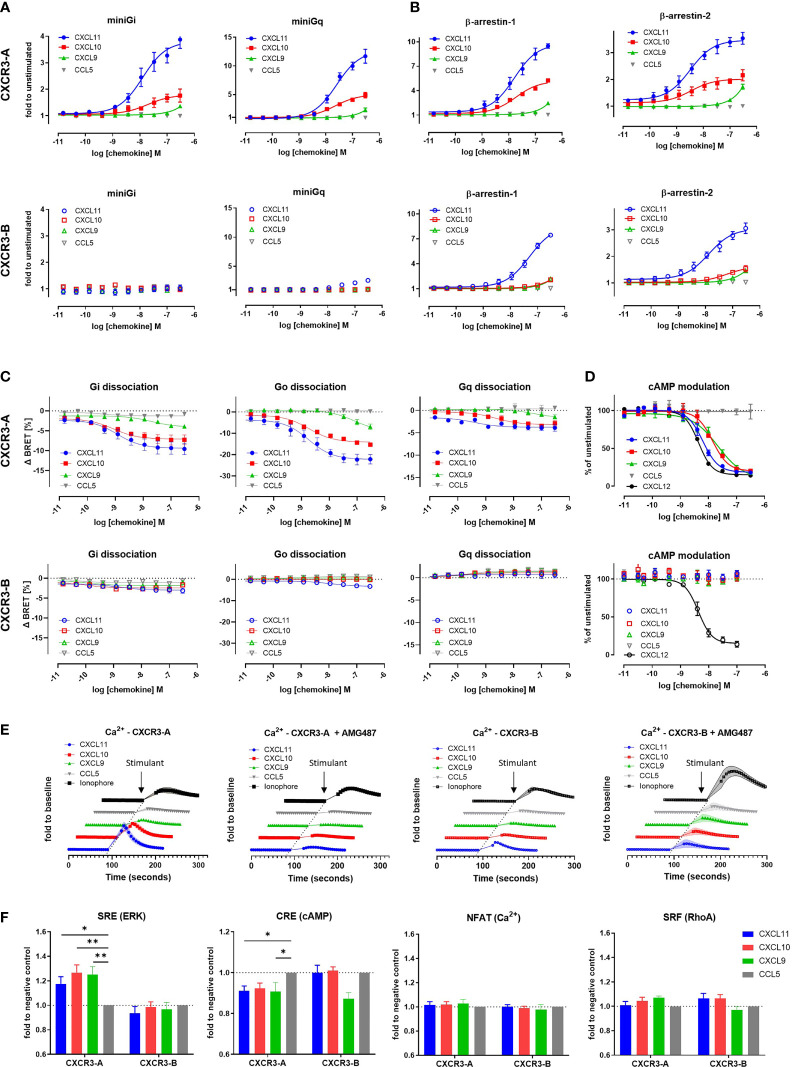
The CXCR3-B isoform does not induce G protein-mediated signaling. **(A)** miniG protein or **(B)** β-arrestin recruitment towards CXCR3-A and CXCR3-B in response to CXCL11, CXCL10, CXCL9 and the negative control CCL5 monitored by NanoBiT-based assay. **(C)** Heterotrimeric G protein dissociation upon CXCR3-A or CXCR3-B stimulation with CXCL11, CXCL10, CXCL9 and CCL5 monitored by NanoBRET. Concentration-response relationships for the alpha subunits G_i2_, G_o_ and G_q_ are expressed as ΔBRET. **(D)** CXCR3-A- and CXCR3-B-driven reduction of intracellular cAMP levels, in response to chemokine ligands. CXCL12-induced effect mediated by the endogenously expressed CXCR4 was used as positive control. **(E)** CXCR3-A- and CXCR3-B-mediated intracellular calcium mobilization in response to chemokines in the absence or presence of CXCR3 antagonist AMG487 (1 µM) monitored by NanoBiT assay. Calcium ionophore A23187 (1 µM) was used as receptor-independent positive control. **(F)** Comparison of CXCR3 isoform-mediated downstream signaling events using the nanoluciferase-dependent response elements SRE, CRE, NFAT and SRF in response to the chemokines CXCL11, CXCL10, CXCL9 and CCL5 (100 nM). All assays were conducted in HEK293T cells. Data points represent mean ± SEM of three independent experiments. *p < 0.05 and **p < 0.01 by Kruskal-Wallis with two-sided Dunn’s test.

To evaluate and compare the functionality and signaling capacity of CXCR3-A and CXCR3-B isoforms, we first investigated their ability to interact with miniG proteins and β-arrestins in response to their shared ligands CXCL11, CXCL10, and CXCL9 using a nanoluciferase complementation-based (NanoBiT) assay. CXCR3-A was able to recruit miniG_i_ and miniG_q_ proteins after stimulation with all three chemokines. In stark contrast, CXCR3-B showed no miniG_i_ recruitment while miniG_q_ recruitment was nearly abolished (**Figure 1A**). Both CXCR3-A and CXCR3-B failed to recruit miniG_s_ and miniG_12/13_ proteins (**Supplementary Figure 2**). Next, we monitored the recruitment of β-arrestins towards both CXCR3 isoforms upon chemokine stimulation. CXCL11 or CXCL10 induced β-arrestin-1 and β-arrestin-2 recruitment to CXCR3-A, with CXCL11 having stronger potency and efficacy compared to CXCL10 (**Figure 1B** and **Supplementary Table 1**). Interestingly, although G protein interaction was severely impaired, CXCR3-B efficiently interacted with β-arrestin-1 and β-arrestin-2 upon stimulation with CXCL11 and a weak recruitment for CXCL10 was detected. CXCL9 was only able to induce a slight β-arrestin recruitment towards both CXCR3 isoforms at the highest concentration tested (**Figure 1B**). These results were confirmed using the same assays in U-87 MG cellular background as well as using NanoBRET-based approaches (**Supplementary Figure 2**).

The lack of CXCR3-B coupling to G proteins was further investigated using a NanoBRET assay monitoring heterotrimeric G protein dissociation following chemokine-induced receptor activation. CXCR3-A stimulation by its ligands triggered the dissociation of G_αi/o_ and G_αq_ proteins from the G_βγ_ dimer, albeit a weaker effect was observed for the latter. In contrast, CXCR3-B displayed no G_i_ and G_q_ activation and a severe impairment of G_o_ for CXCL10 and CXCL9. CXCL11 triggered slight activation of G_i/o_ protein at high chemokine concentration (**Figure 1C**). No other G protein subtype activation could be measured for either CXCR3 isoform (**Supplementary Figure 3**). These results support the data obtained for miniG protein recruitment and further confirm the impairment of CXCR3-B coupling to G proteins.

To corroborate these observations, we investigated the modulation of two downstream G protein signaling secondary messengers: cAMP and calcium. cAMP modulation was monitored using a firefly luciferase-based Glo biosensor. Upon activation with CXCL11, CXCL10, CXCL9, we detected a concentration-dependent decrease in cAMP for CXCR3-A, confirming G_i/o_ protein activation. No cAMP modulation was however detected for CXCR3-B (**Figure 1D**) nor the untransfected cells after stimulation with CXCL11, CXCL10 or CXCL9 (**Supplementary Figure 3**). The positive control CXCL12 was able to reduce cAMP levels, by acting on endogenously expressed CXCR4, attesting to the assay functionality. Intracellular calcium mobilization was investigated using a NanoBiT-based calmodulin–MYLK2S assay to further characterize signaling abilities of the receptors. CXCR3-A induced a calcium flux in response to all three of its cognate chemokines. These calcium fluxes could be inhibited using the CXCR3 antagonist AMG487, confirming the CXCR3-mediated calcium measurements (**Figure 1E**). In contrast, CXCR3-B only triggered a weak calcium flux in response to CXCL11 and no response to CXCL10 or CXCL9. Similar results were obtained by using the ratiometric fluorescent calcium indicator Indo-1 (**Supplementary Figure 3**). These experiments confirmed CXCR3-B’s inability to trigger efficient downstream G protein signaling (**Figures 1D, E**), which is in agreement with the impaired G_i/o_ activation.

Finally, the ability of the CXCR3-binding chemokines to trigger later downstream signaling events was also examined by monitoring the activation of various transcriptional response elements (RE). Stimulation of CXCR3-A by CXCL11, CXCL10 and CXCL9 led to an increase in the activation of MAPK/ERK-dependent response element SRE, pointing to the involvement of ERK signaling, as opposed to CXCR3-B that showed no modulation of this pathway (**Figure 1F**). The specific activation of G_i_ or G_s_ and subsequent cAMP modulation was monitored by CRE inhibition. A slight decrease in signal for CXCR3-A was observed after stimulation with CXCL11, CXCL10, and CXCL9, while no difference in signal could be seen for CXCR3-B with the exception of CXCL9 that showed a more pronounced but statistically not significant decrease (**Figure 1F**). We also examined prolonged increase of intracellular calcium ions and G_12/13_ protein-specific RhoA-mediated signaling using NFAT and SRF response elements, respectively, which showed no differences in the signal measured in response to CXCR3 chemokines for both isoforms compared to the negative control CCL5 (**Figure 1F**).

Together, these data point to an altered ability of the CXCR3-B isoform to couple to and activate G proteins and their downstream signaling events, while preserving the ability to recruit β-arrestins upon chemokine stimulation.

### 3.2 CXCR3-B Has a Different Cellular Localization Compared to CXCR3-A

Based on the observations that CXCR3-B efficiently recruits β-arrestins despite the impaired G protein signaling, we examined whether it shows other properties characteristic of atypical chemokine receptors. We first evaluated the basal cellular distribution of both CXCR3 isoforms and compared it to that of ACKR2 and ACKR3, two receptors of the ACKR family reported to be mostly located in intracellular vesicles under basal conditions (13).

**Figure 2:**
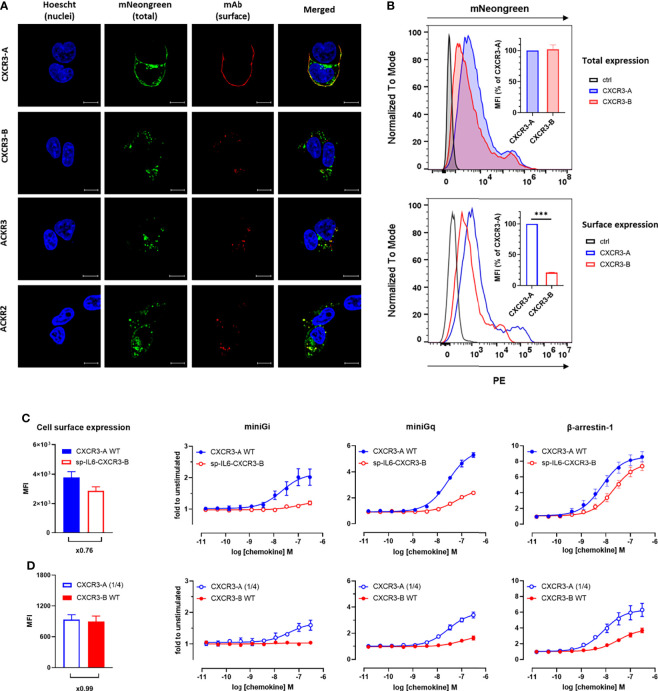
CXCR3-B shows intracellular localization and does not cycle in basal conditions but is mobilized to the cell membrane upon chemokine stimulation. **(A)** Receptor cellular distribution in basal conditions visualized by confocal fluorescent microscopy using mNeonGreen-fused CXCR3-A, CXCR3-B, ACKR3 and ACKR2 for total expression and receptor-specific mAbs (clones 1C6, 8F11-M16 and 196124, respectively) for surface expression. Pictures are representative of 12 acquired images from three independent experiments. **(B)** Receptor expression monitored by flow cytometry in cells transiently transfected with vectors encoding CXCR3-A or CXCR3-B C-terminally tagged with mNeonGreen or empty vectors (ctrl). Total expression was evaluated as mNeonGreen (upper panel) and surface expression by CXCR3-specific mAb (clone 1C6). Both are quantified as mean fluorescent intensity (MFI) and expressed as percentage of CXCR3-A (inset). Data shown are representative of three independent experiments and for inset, mean ± SEM of three independent experiments. ***p < 0.0001 by unpaired t test. **(C, D)** miniG protein or β-arrestin recruitment towards cell surface-controlled levels of CXCR3-A and CXCR3-B monitored by NanoBiT. Equivalent surface expression levels for the two isoforms were obtained by the addition of human interleukin-6-derived signal peptide to facilitate CXCR3-B export to the plasma membrane (sp-IL6-CXCR3-B) **(C)** or by reducing the amount of CXCR3-A-encoding vector used for transfection (CXCR3-A (1/4)) **(D)**. Cell surface expression of the receptor variants was assessed by flow cytometry with CXCR3-specific mAb (clone 1C6). CXCL11-induced miniGi, miniGq or β-arrestin-1 recruitment to CXCR3-A WT and sp-IL6-CXCR3-B **(C)** or CXCR3-A (1/4) and CXCR3-B WT **(D)**.

The cellular distribution of CXCR3 isoforms, was first visualized by confocal fluorescent microscopy using receptors C-terminally fused to the mNeonGreen fluorescent protein and a receptor-specific antibody to detect their presence at the plasma membrane. In line with previous reports (18), CXCR3-A was mainly present at the cell surface as revealed by the strong co-localization of mNeonGreen and the CXCR3-specific antibody (**Figure 2A**). In contrast, CXCR3-B showed a more pronounced intracellular distribution and a reduced fluorescent signal at the plasma membrane, which was reminiscent of the intracellular localization of ACKR3 and ACKR2 (**Figure 2A**). The differences in cellular distribution of the two CXCR3 isoforms were further confirmed by flow cytometry. Indeed, despite a similar total expression level of the two isoforms, CXCR3-A showed greater surface expression compared to CXCR3-B (**Figure 2B**). Consistently, results obtained with the high-affinity nanoluciferase complementation-based HiBiT assay revealed that only 45% of total CXCR3-B was found at the cell surface under basal conditions, contrasting with the almost exclusive surface localization of CXCR3-A (**Supplementary Figure 4**).

These observations prompted us to investigate further the accountability of the different surface expression levels of the two isoforms in their abilities to recruit cellular effectors. To do so, we adopted two strategies. We first generated a CXCR3-B variant preceded by the signal peptide of human interleukin-6 (sp-IL6) known to facilitate receptor export to the plasma membrane. Despite the similar cell surface expression of the two variants (**Figure 2C**, left panel), only low levels of miniGi or miniGq recruitment could be observed for sp-IL6-CXCR3-B, while its ability to recruit β-arrestin-1 was comparable to CXCR3-A (**Figure 2C**). In the second opposite approach, equivalent surface expression levels for the two isoforms were achieved by reducing four times the amount of CXCR3-A-encoding DNA used in transfection (**Figure 2D**, left panel). In these conditions, CXCR3-B still showed drastically impaired G protein coupling compared to only slightly diminished CXCR3-A activation (**Figure 2D**). These results show that although β-arrestin interaction and receptor-mediated G-protein activation are to a certain extent influenced by receptor surface expression, the different subcellular localization is not sufficient to fully explain the divergent properties of CXCR3-A and CXCR3-B.

### 3.3 CXCR3-B Does Not Cycle in Basal Conditions but Is Mobilized to the Cell Membrane Upon Chemokine Stimulation

The atypical subcellular distribution of CXCR3-B is reminiscent of that of ACKRs, which play an important role in regulating the extracellular chemokine availability for classical receptors. Chemokine binding to ACKRs may induce their internalization, but some receptors, like ACKR3, were shown to continuously cycle between the intracellular compartments and the cell surface, independently of ligand stimulation (43, 44).

**Figure 3:**
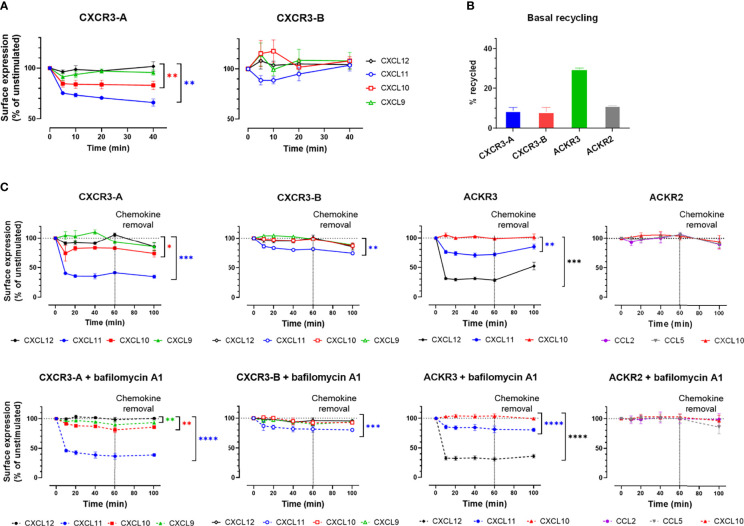
CXCR3-B is mobilized to the cell membrane upon chemokine stimulation but does not cycle in basal conditions. **(A, C)** Cell surface redistribution of CXCR3-A and CXCR3-B after stimulation with chemokines (100 nM) monitored by **(A)** surface nanoluciferase complementation (HiBiT) or **(C)** flow cytometry in the presence or absence of bafilomycin A1. All assays were conducted in HEK293T cells. Results are expressed as percentage of receptor surface expression in basal conditions (100%) and represent mean ± SEM of three independent experiments. *p < 0.05, **p < 0.01, ***p < 0.001 and ****p < 0.0001 by repeated measures one-way ANOVA with Bonferroni correction. **(B)** CXCR3-A, CXCR3-B, ACKR3 and ACKR2 basal cycling after extracellular epitope shaving by proteinase K. Results are expressed as percentage of cell surface expression after proteinase K treatment.

We therefore investigated CXCR3 cycling in the absence and presence of chemokines. Basal receptor cycling was first evaluated in flow cytometry following extracellular epitope cleavage by proteinase K, by monitoring receptor replenishment at the plasma membrane in the presence of cycloheximide, a *de-novo* protein synthesis inhibitor. Two distinct trends could be identified for the set of chemokine receptors tested. Both CXCR3 isoforms and ACKR2 showed an approximately 10%-increase of receptor cell surface expression, while ACKR3 demonstrated a more pronounced increase of 30% (**Figure 3B** and **Supplementary Figure 4**). This suggests that similarly to CXCR3-A and ACKR2, CXCR3-B shows limited cycling from the intracellular compartment to the plasma membrane in the absence of chemokines.

Chemokine-induced internalization and recycling was then assessed for the two CXCR3 isoforms, first by flow cytometry (**Figure 3C** and **Supplementary Figure 4**). CXCL11 and CXCL10 induced the internalization of CXCR3-A after 10-minute stimulation, with CXCL11 reducing the receptor surface expression by 60% and CXCL10 by 20%, while CXCL9 had no impact on receptor internalization (**Figure 3C**). In contrast, for CXCR3-B, CXCL11 induced only about 20% net internalization, while CXCL10 and CXCL9 had no effect (**Figure 3C**). Overall ACKR2 surface expression was not modified by chemokine stimulation, reminiscent of CXCR3-B behavior, whereas ACKR3 stimulation with CXCL12 and CXCL11 led to the internalization of approximately 70% and 25% of the receptor present at the plasma membrane, respectively (**Figure 3C**). In addition, receptor surface expression was evaluated 40 minutes after chemokine removal. None of the CXCR3 isoforms nor ACKR2 recycled back to the cell surface in the presence or absence of the V-ATPase inhibitor bafilomycin A1, suggesting an absence of rapid recycling following ligand stimulations (**Figure 3C**) in contrast to ACKR3, for which bafilomycin A1-sensitive recovery at the plasma membrane could be detected (34, 45).

The limited level of CXCR3-B internalization, compared to CXCR3-A, upon stimulation was further studied using a highly sensitive cell surface detection approach based on the HiBiT nanoluciferase complementation technology. Cells expressing N-terminally HiBiT-tagged CXCR3-A or CXCR3-B were stimulated with chemokines and the remaining membrane receptors were quantified by adding soluble LgBiT protein. A decrease in CXCR3-A receptor level at the cell surface was induced by CXCL10 and CXCL11 (**Figure 3A**) and reflected their respective potencies in β-arrestin recruitment assays (**Figure 1B** and **Supplementary Table 1**). In contrast, although an initial reduction of CXCR3-B levels was observed in response to CXCL11, a gradual replenishment of the receptor at the cell surface was then measured. Moreover, an immediate but not statistically significant increase in surface CXCR3-B was triggered by CXCL10 and CXCL9, suggesting a rapid transport of a part of the intracellular receptor pool to the plasma membrane (**Figure 3A**) as recently described for ACKR2 following ligand stimulation (35).

Altogether, these results confirm that CXCR3-A and CXCR3-B have different cellular distribution patterns under basal and ligand-induced conditions. CXCR3-A exhibits a classical chemokine receptor profile, with a more pronounced cell surface expression and chemokine-induced internalization, while CXCR3-B resides inside the cell in basal conditions and generally shows a slower internalization upon ligand stimulation and a mobilization to the plasma membrane upon stimulation that are reminiscent of the profile observed for ACKR2 (35).

### 3.4 Both CXCR3 Isoforms Mediate Efficient Uptake of Endogenous Chemokines

As CXCR3-B is unable to induce canonical G protein signaling events in response to ligand binding, while maintaining its ability to recruit β-arrestin, we next examined whether this receptor was able to internalize CXCR3 ligands.

**Figure 4:**
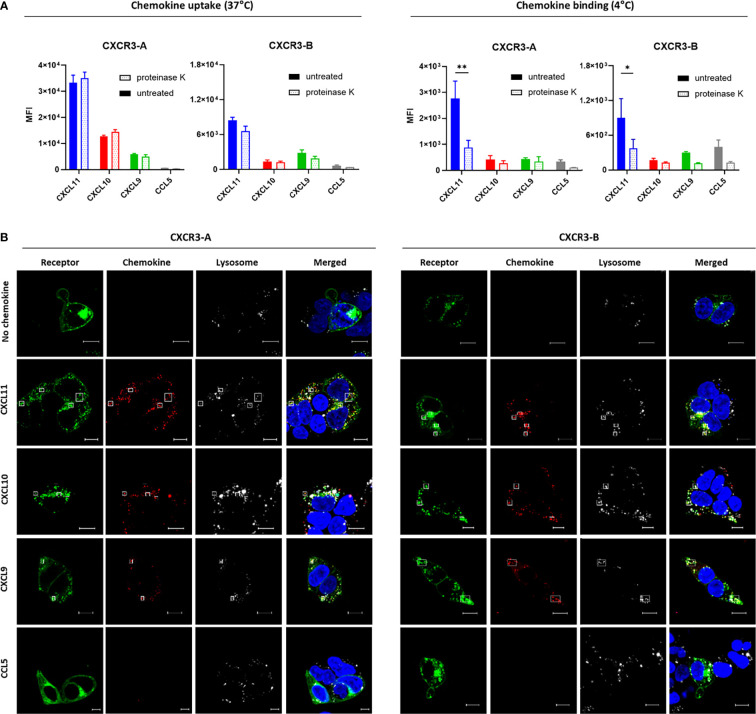
Both CXCR3 isoforms can mediate chemokine uptake. **(A)** Intracellular accumulation (37°C) and cell surface binding (4°C) of Alexa Fluor 647-labeled CXCL11, CXCL10, CXCL9 and CCL5 (33 nM) monitored by flow cytometry in CXCR3-A- and CXCR3-B-expressing cells. After 1-hour incubation cell surface-bound chemokines were removed with proteinase K treatment. Results represent mean ± SEM of three independent experiments. *p < 0.05, **p < 0.01 by two-way ANOVA with Tukey’s *post hoc* analysis. **(B)** CXCR3-A- and CXCR3-B-driven uptake of Alexa Fluor 647-coupled chemokines visualized by confocal fluorescent microscopy. Cells transiently expressing CXCR3-A or CXCR3-B fused with mNeonGreen (green) were incubated for 2 hours with chemokines (100 nM) (red). Lysosomes and nucleic DNA were stained using LysoTracker™ Red DND-99 (white) and Hoechst 33342 (blue), respectively. Pictures are representative of 12 acquired images from three independent experiments. Scale bar: 10 µm. All assays were conducted in HEK293T cells.

We first investigated the uptake of all CXCR3 ligands coupled to Alexa Fluor 647 by flow cytometry. CXCL11, CXCL10 and CXCL9 uptake was detected for both isoforms, albeit with reduced intensities for CXCR3-B (**Figure 4A**), which may reflect its lower expression level at the plasma membrane. No uptake was observed with the irrelevant chemokine CCL5 labeled with the same fluorophore. Chemokine targeting to intracellular compartments was confirmed with the use of proteinase K, a non-selective protease allowing to remove remaining cell surface-bound chemokines, as illustrated by the reduced fluorescence signal in non-internalizing conditions (4°C) for both CXCR3-A and CXCR3-B-expressing proteinase K-treated cells. In contrast, this treatment had no impact on the signal detected following chemokine incubation in internalizing conditions (37°C) for the two CXCR3 isoforms, strongly pointing to chemokine uptake following receptor activation.

Receptor-dependent internalization of fluorescently labeled chemokines was then analyzed by confocal microscopy. Cells expressing CXCR3-A or CXCR3-B internalized their related chemokines (**Figure 4B**) in contrast to non-transfected cells (**Supplementary Figure 4**). Lysotracker, a fluorescent dye for labeling and tracking acidic organelles in living cells, confirmed the intracellular co-localization of the receptor and the internalized chemokines, hinting towards their degradation. Furthermore, a noticeable change in receptor subcellular distribution could be observed for CXCR3-A after stimulation, but not for CXCR3-B, corroborating the different receptor internalization profiles described above.

These data demonstrate that CXCR3-B is able to mediate the uptake of CXCR3 ligands from the extracellular space and to address them to intracellular compartment despite its inability to trigger efficient G protein signaling.

### 3.5 The N-Terminal Extension of CXCR3-B Does Not Modify Chemokine Selectivity and Binding Mode

The extracellular N-terminal domain of chemokine receptors plays an important role in chemokine binding and selectivity (46). The presence of the unique extended CXCR3-B N terminus and its impact on the ability of the receptor to efficiently couple to G proteins prompted us to investigate other receptor properties such as selectivity and activation mode.

**Figure 5:**
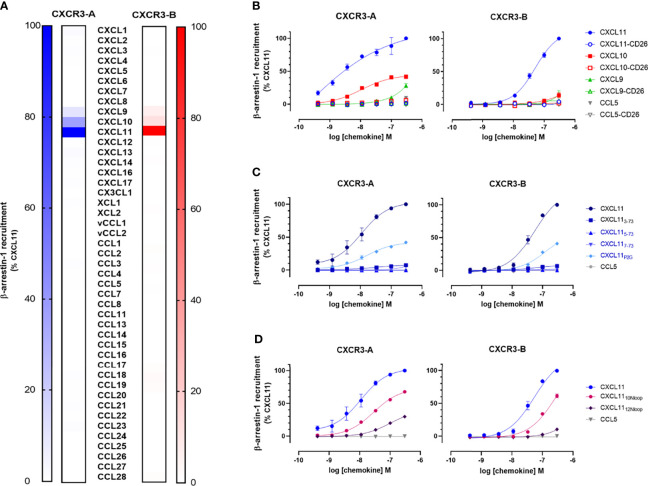
N-terminal extension of CXCR3-B does not change the receptor selectivity and chemokine binding mode. **(A)** β-arrestin-1 recruitment to CXCR3-A (blue color scale) and CXCR3-B (red color scale) in response to all known human and two viral chemokines (100 nM). **(B)** Impact of chemokine N-terminal processing by dipeptidyl peptidase 4 (DPP4/CD26) on the chemokine-induced β-arrestin-1 recruitment to CXCR3 isoforms. **(C)** Impact of N-terminal truncation and P2G-mutation on CXCL11-induced β-arrestin-1 recruitment to CXCR3-A and CXCR3-B. **(D)** Recruitment of β-arrestin-1 to CXCR3-A and CXCR3-B in response to CXCL11 Nloop chimeras. All data were generated using NanoBiT-based assays in HEK293T cells and are expressed as percentage relative to maximum of the full agonist CXCL11 at 100 nM **(A)** or 300 nM **(B–D)**. Data represent mean ± SEM of three independent experiments.

To this end, we first undertook a β-arrestin-1 recruitment screening of the 43 human chemokines (24 CCLs, 16 CXCLs, 2 XCLs, and 1 CX3CL) and 2 viral chemokines (vCCL1 and vCCL2) towards the CXCR3 isoforms aiming at evaluating the possible impact of the extended CXCR3-B N terminus on ligand selectivity. This systematic interaction assessment did not allow to identify new endogenous agonist chemokines for any of the two isoforms (**Figure 5A**). Noteworthy, we could not confirm the activity of the orphan chemokine CXCL4 proposed in some studies as a CXCR3-B agonist (18, 47). Surprisingly, we observed that the viral chemokine vCCL2 showed antagonistic properties towards both CXCR3 isoforms, an interaction that has not been described previously (**Supplementary Figure 5**) (48).

Atypical chemokine receptors may have different recognition determinants and activation modes compared to classical receptors. Notably, ACKR3, which acts as a scavenger for CXCL11, was shown to be insensitive to chemokine N loop substitutions and cleavage by the dipeptidyl peptidase 4 (CD26), which, by removing the first two residues turns agonist CXC chemokines into antagonist for a great majority of receptors (49). Therefore, we next investigated the ability of chemokines with N-terminal substitutions and progressive truncations or modified N loops to activate both CXCR3 isoforms. The removal of the first two residues of CXCL11, CXCL10, and CXCL9 by CD26 exopeptidase abolished their activity towards both CXCR3 isoforms (**Figure 5B**). These results were confirmed with recombinant CXCL11 lacking the first two residues (CXCL11_3-73_) or bearing further N-terminal truncations (CXCL11_5-73_ and CXCL11_7-73_) (**Figure 5C**). Similarly, proline-to-glycine substitution at position 2 (CXCL11_P2G_), known to improve CXCL11 potency towards ACKR3 (31), had a negative impact on the chemokine’s ability to activate the two CXCR3 isoforms. Finally, CXCL11 chimeras with CXCL12 or CXCL10 N loop substitutions (CXCL11_12Nloop_ and CXCL11_10Nloop_), had a reduced potency and efficacy towards both isoforms, CXCL11_12Nloop_ being the most affected (**Figure 5D** and **Supplementary Table 2**).

Taken together, these results demonstrate similar effect of chemokine modifications on receptor interactions for the two isoforms, suggesting a shared recognition and activation mode.

### 3.6 The N-Terminal Extension of CXCR3 Is Responsible for Its Intracellular Localization and Associated Lack of G Protein Coupling

Considering the impact of the CXCR3-B N-terminal extension on the receptor localization and coupling to G proteins, we investigated whether the entire extension is required to observe these effects and whether they are specific to CXCR3.

**Figure 6:**
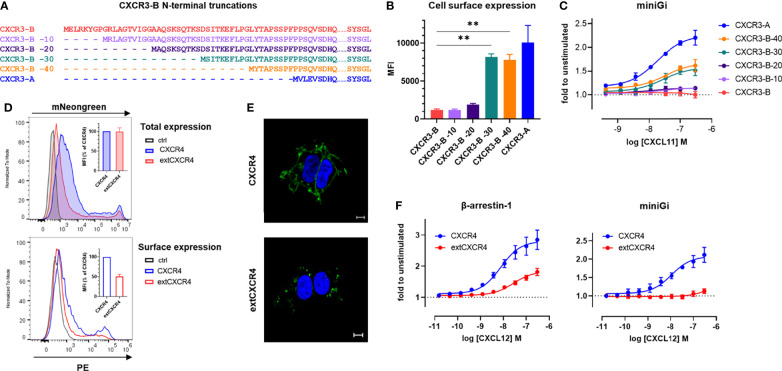
The N-terminal extension of CXCR3-B is responsible for its intracellular localization and lack of G protein coupling. **(A)** Amino acid sequence comparison of CXCR3-B variants with progressive N-terminal truncations (CXCR3-B -10, CXCR3B -20, CXCR3-B -30, CXCR3B -40). **(B)** Cell surface expression of CXCR3-B bearing progressive N-terminal truncations determined by flow cytometry using the CXCR3-specific mAb 1C6. **(C)** CXCL11-induced miniGi protein recruitment to N-terminally truncated CXCR3-B determined by NanoBiT-based assay. **(D)** Receptor expression monitored by flow cytometry in cells transiently transfected with vectors encoding CXCR4 or extCXCR4 C-terminally tagged with mNeonGreen or empty vectors (ctrl). Total expression was evaluated as mNeonGreen (upper panel) and surface expression by CXCR4-specific mAb (clone 12G5). Both are quantified as mean fluorescent intensity (MFI) and expressed as percentage of CXCR4 (inset). **(E)** CXCR4 and extCXCR4 cellular distribution visualized by confocal fluorescent microscopy in cells transiently expressing mNeonGreen-fused CXCR4 or extCXCR4. HEK293T cells were used as cellular background for all the panels. Pictures are representative of 10 acquired images from two independent experiments. Scale bar: 10 μm. **(F)** CXCL12-induced β-arrestin-1 or miniGi recruitment to CXCR4 and the chimeric extCXCR4 monitored with NanoBiT-based assays. For **(A–D, F)** data represent mean ± SEM of three independent experiments. For **(B)** **p < 0.01 by ordinary one-way ANOVA with Tukey’s *post hoc* analysis.

Progressive truncations in the N terminus of CXCR3-B were introduced (**Figure 6A**) and the membrane expression as well as the ability of the resulting receptors to interact with miniG proteins were evaluated. The N-terminal truncations resulted in a gradual increase of the receptor presence at the cell surface, the most significant increment being observed upon the removal of approximately half of the extension (CXCR3-B -30), reaching a plateau that was nevertheless lower than for the surface expression of CXCR3-A (**Figure 6B**). A matching trend in G protein coupling of the progressively truncated receptors was also observed (**Figure 6C** and **Supplementary Table 3**).

Finally, we showed that a similar effect on G protein coupling can be achieved by inserting the CXCR3-B extension in another classical chemokine receptor, CXCR4. Indeed, the engraftment of the N-terminal extension of CXCR3-B to the N terminus of CXCR4 (extCXCR4) resulted in a loss of G_i/o_ protein coupling, while the ability to recruit β-arrestin-1 in response to CXCL12 was conserved (**Figure 6F**). The flow cytometry and confocal microscopy analysis of surface expression and cellular distribution also demonstrated that just like for CXCR3 isoform, extCXCR4 resides more intracellularly, in dot like structures, than WT CXCR4 (**Figures 6D, E**).

These observations demonstrate that the extended CXCR3-B N terminus impacts the classical receptor cellular localization and G protein coupling, while it has a limited effect on the its ability to recruit β-arrestin and mediate chemokine uptake, giving CXCR3-B the attributes of an atypical receptor, despite being encoded by the same gene as CXCR3-A.

## 4 Discussion

CXCR3-B isoform is generated following alternative splicing of the *CXCR3* gene and presents an extended N terminus compared to CXCR3-A, the most studied isoform of the receptor.

CXCR3-B isoform is an elusive and enigmatic chemokine receptor for which divergent functional and signaling results exist (18, 20, 21, 50). CXCR3-B has been suggested to be biased towards β-arrestins (20, 21) but the mechanisms for this bias and the atypical nature of the receptor have not been established. We therefore undertook an in-depth molecular characterization of CXCR3-B to provide signaling and mechanistic insights into its biology and function in comparison to CXCR3-A. Our study validates the β-arrestin bias of CXCR3-B and reveals that it shows several attributes characteristic of the atypical chemokine receptor family. This suggests that CXCR3-B may act as a scavenger for the CXCR3-binding chemokines, possibly explaining its opposite biological effects compared to CXCR3-A.

A common characteristic of ACKRs is their inability to trigger downstream G protein-dependent signaling events upon agonist stimulation. Instead, most ACKRs recruit β-arrestins to mediate receptor internalization, although recent reports suggest that the presence of β-arrestins is not essential for this function (9–12). Our study shows that, similarly to ACKRs, CXCR3-B is unable to efficiently activate G proteins and the related downstream signaling pathways following chemokine stimulation, as opposed to CXCR3-A. However, CXCR3-B retained its ability to recruit β-arrestins and to mediate chemokine uptake. These observations are in line with other reports showing that the two CXCR3 isoforms differentially affect downstream pathways (20, 21, 51).

CXCR3-B was proposed to trigger cellular signaling such as ERK phosphorylation (20, 21), although, it remains unclear by which effectors this pathway may be activated. Here, we show, using methods that allow to monitor directly protein-protein interactions, that no G proteins, nor related signaling events, are activated by CXCR3-B, further underscoring its atypical nature.

We also show that the intracellular localization of CXCR3-B may be attributed to its N terminal extension. The N-terminal domain of chemokine receptors is known to play an important role in chemokine binding (46, 52, 53), and its elongation with various N-terminal tags or reporter proteins has already been shown to impact the receptor biology and pharmacology, requesting in some cases the addition of exogenous proximal signal peptide to ensure a proper export to the plasma membrane. Likewise, the extension of CXCR3-B drastically changes the receptor cellular distribution, limiting its presence at the cell surface. Our data also show that under basal conditions, CXCR3-B does not cycle between the plasma membrane and intracellular compartments. In contrast, CXCR3-B intracellular pool is mobilized to the cell surface upon stimulation. Depending on the chemokine, different directionalities of CXCR3-B plasma membrane level modulation were observed, which may result from their respective potency to induce receptor internalization. Indeed, CXCL10 and CXCL9, known to be weak CXCR3-internalizing chemokines, induced a net increase of CXCR3-B at the plasma membrane, whereas the more efficacious receptor-internalizing chemokine, such as CXCL11, led to net reduction of the receptor present at the cell surface (54, 55).

Uptake experiments using fluorescently labeled chemokines demonstrated that the overall mobilization of the receptor ultimately results in specific intracellular accumulation of all the three CXCR3 chemokines, albeit with different efficacies compared to CXCR3-A. This scavenging ability was confirmed by confocal microscopy showing co-localization of the receptor with the chemokines in acidic degradation compartments, reminiscent of the behavior of well-characterized ACKRs such as ACKR2, ACKR3 or ACKR4. Moreover, the observation that CXCL9 and CXCL10 are efficiently and specifically taken up by both CXCR3 isoforms, while they show no or reduced ability to induce β-arrestin recruitment towards CXCR3, suggests that β-arrestin-independent mechanisms may mediate the chemokine-induced receptor internalization and trafficking, as recently suggested for other ACKRs (11, 12).

Although biophysical studies have suggested that small molecules can induce different conformational changes in CXCR3-A and CXCR3-B (56), our results generated using chimeric or truncated chemokines indicate that the two CXCR3 isoforms share the same chemokine binding and activation modes, suggesting that ligand–receptor interactions are not at the origin of the impaired G protein coupling of CXCR3-B. On the other hand, progressive truncation of the N-terminal extension of CXCR3-B partially restored surface expression and the ability of the receptor to couple to G proteins. However, we showed that even when expressed at equivalent levels at the cell surface CXCR3-B still shows a reduced ability to interact with G protein. This suggests that the change in the cellular localization of the receptor and the impact of the N-terminal extension on the activated receptor conformation are the two main drivers of this shift of signaling properties, most probably limiting its ability to efficiently activate G proteins, while preserving its ability to cycle and internalize chemokines, consequently conferring to CXCR3-B attributes of atypical chemokine receptors.

Indeed, the absence of G protein signaling, the predominantly intracellular localization and the ability to actively take up chemokines are three major attributes of atypical chemokine receptors (2). We therefore propose that CXCR3-B could be regarded as an atypical chemokine receptor that modulates the bioavailability of CXCR3 chemokines thereby regulating the activation and cellular effects of CXCR3-A. Interestingly, in inflammatory conditions and in the tumor environment, CXCR3-A and CXCR3-B were reported to display opposing effects, which could be explained, in light of the present study, by their distinct function as signaling and scavenging receptors, respectively. Similar contrasting functions for CXCR3 have been documented in zebrafish where the gene is triplicated. In this organism, Cxcr3.2 and Cxcr3.3 copies, both expressed on macrophages, have been shown to coordinate their migration during bacterial infection by functioning antagonistically. Cxcr3.3 shows atypical properties and is not able to elicit G protein-mediated signaling upon ligand stimulation. It was therefore suggested to attenuate the signaling of Cxcr3.2 by scavenging the ligands the two receptors share (57). Of note, other natural human isoforms of chemokine receptors displaying N- or C-terminal extensions and showing altered biology have also been described for CXCR4, CCR9, CCR2, CX3CR1, but also for other GPCR families (58–61).

In conclusion, this study provides signaling and mechanistic insights into the differences of the CXCR3 isoforms that may explain their opposite effects. Our data indicate a strong atypical profile for CXCR3-B with features such as complete absence of G protein coupling, intracellular localization and chemokine uptake capacities, which can be attributed to its N-terminal extension. Additional investigations are required to get a better understanding of this enigmatic receptor and to be able to develop molecules or antibodies capable to specifically modulate CXCR3-B.

## Data Availability Statement

The original contributions presented in the study are included in the article/**Supplementary Material**. Further inquiries can be directed to the corresponding author.

## Author Contributions

GD’U, NR, JH, MS, and AC designed the study and wrote the manuscript. GD’U, NR, MM, and DA performed the experiments. GD’U, NR, MS, and AC analyzed and interpreted the data. GD’U, NR, MM, TU, and MS generated molecular tools for cellular assays. BFV contributed modified CXCL11 chemokines. TL, BJ, JH, MS, and AC supervised the overall study. All authors approved the manuscript.

## Funding

This study was supported by the Luxembourg Institute of Health (LIH), Luxembourg National Research Fund (Pathfinder “Interceptor” 19/14260467, INTER/FWO “Nanokine” grant 15/10358798, INTER/FNRS grants 20/15084569, and PoC “Megakine” 19/14209621), F.R.S.-FNRS-Télévie (grants 7.4593.19, 7.4529.19 and 7.8504.20) and the European Cooperation in Science and Technology (COST) Action CA18133 European Research Network on Signal Transduction (ERNEST). NR and MM are the Luxembourg National Research Fund PhD fellows (PRIDE-11012546 “NextImmune” and AFR-11274579 grants). GD’U is a F.R.S.-FNRS-Télévie fellow (grant 7.4529.19). AC and MS are part of the Marie Skłodowska-Curie Innovative Training Network ONCORNET2.0 “ONCOgenic Receptor Network of Excellence and Training” (MSCA-ITN-2020-ETN). JH is a F.R.S-FNRS senior research associate and was supported by F.R.S.-Research Project (PDRT.0111.19). DA is a Télévie fellow (7454719F). This work was conducted as part of the MEGAKINE project, which has received funding from the EU Horizon 2020 research and innovation program under the Marie Skłodowska-Curie grant agreement No 896183.

## Conflict of Interest

Author TL was employed by company Confo Therapeutics.

The remaining authors declare that the research was conducted in the absence of any commercial or financial relationships that could be construed as a potential conflict of interest.

## Publisher’s Note

All claims expressed in this article are solely those of the authors and do not necessarily represent those of their affiliated organizations, or those of the publisher, the editors and the reviewers. Any product that may be evaluated in this article, or claim that may be made by its manufacturer, is not guaranteed or endorsed by the publisher.
