# Supplementary material for: The Extended N-Terminal Domain Confers Atypical Chemokine Receptor Properties to CXCR3-B
Source: Front Immunol. 2022 Jun 1;13:868579. doi: 10.3389/fimmu.2022.868579 (PMC9198273; doi:10.3389/fimmu.2022.868579)
Supplement: Supplementary file 1 [file DataSheet_1.pdf]

Supplementary Material

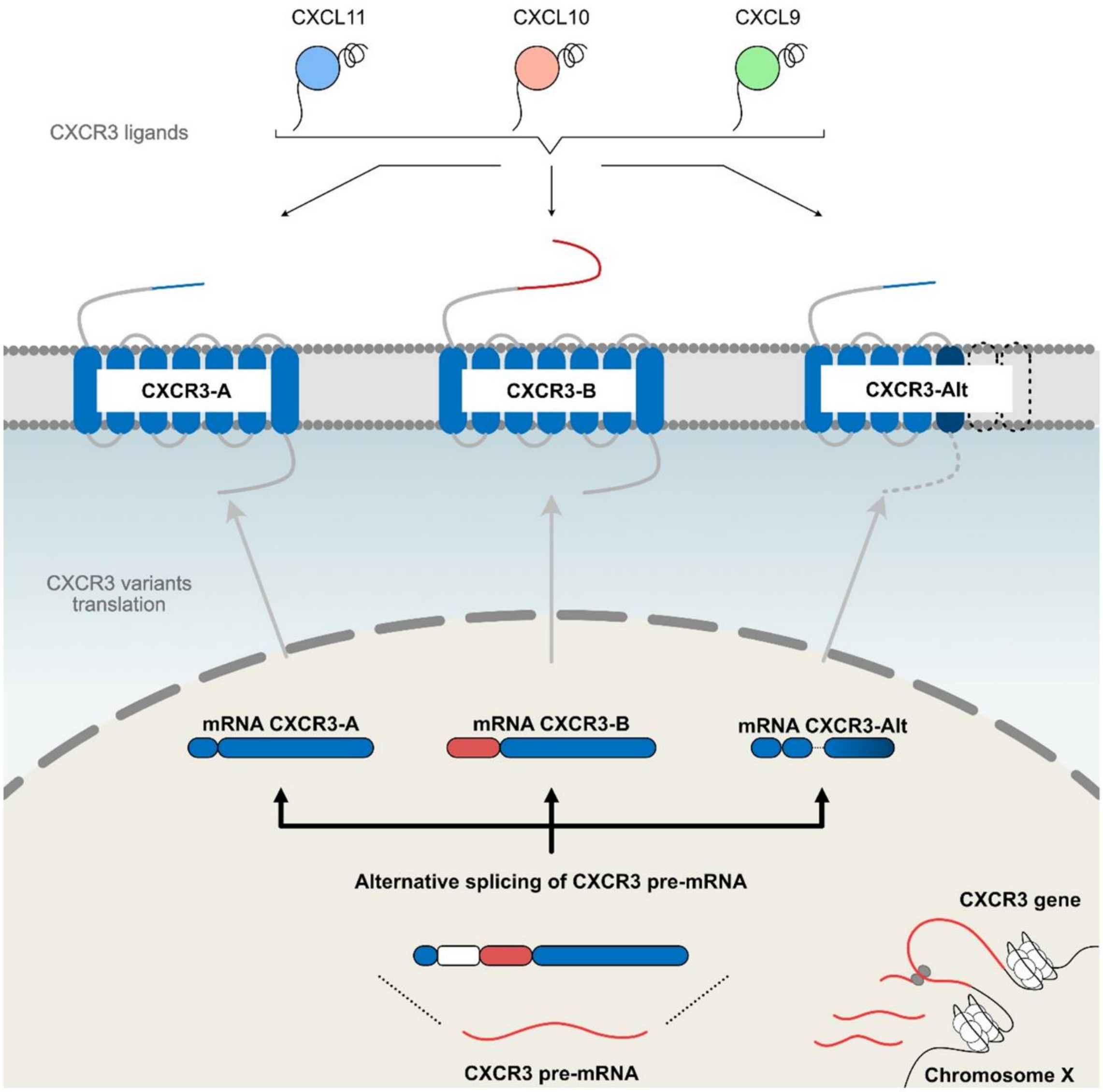

Supplementary figure 1. Overview of the three CXCR3 isoforms. Due to alternative splicing of the pre-mRNA of the *CXCR3* gene, located on chromosome X, three CXCR3 isoforms can be generated. The CXCR3-A isoform is the product of the splicing of the exon 1 and exon 3 within the CXCR3 gene. The assembly of exon 2 and exon 3 results in the CXCR3-B isoform which has an N terminus longer by 47 amino acids compared with CXCR3-A. The removal of the intron, exon 2 and a 337-bp region within the third exon during RNA splicing results in the CXCR3-Alt isoform that comprises the N terminus and the first four transmembrane domains identical to CXCR3-A as well as a possible fifth transmembrane region and a C terminus, which are different from CXCR3-A or CXCR3-B. All CXCR3 isoforms are able to bind three endogenous ligands CXCL9, CXCL10 and CXCL11, each with a different binding affinity and activation potential.

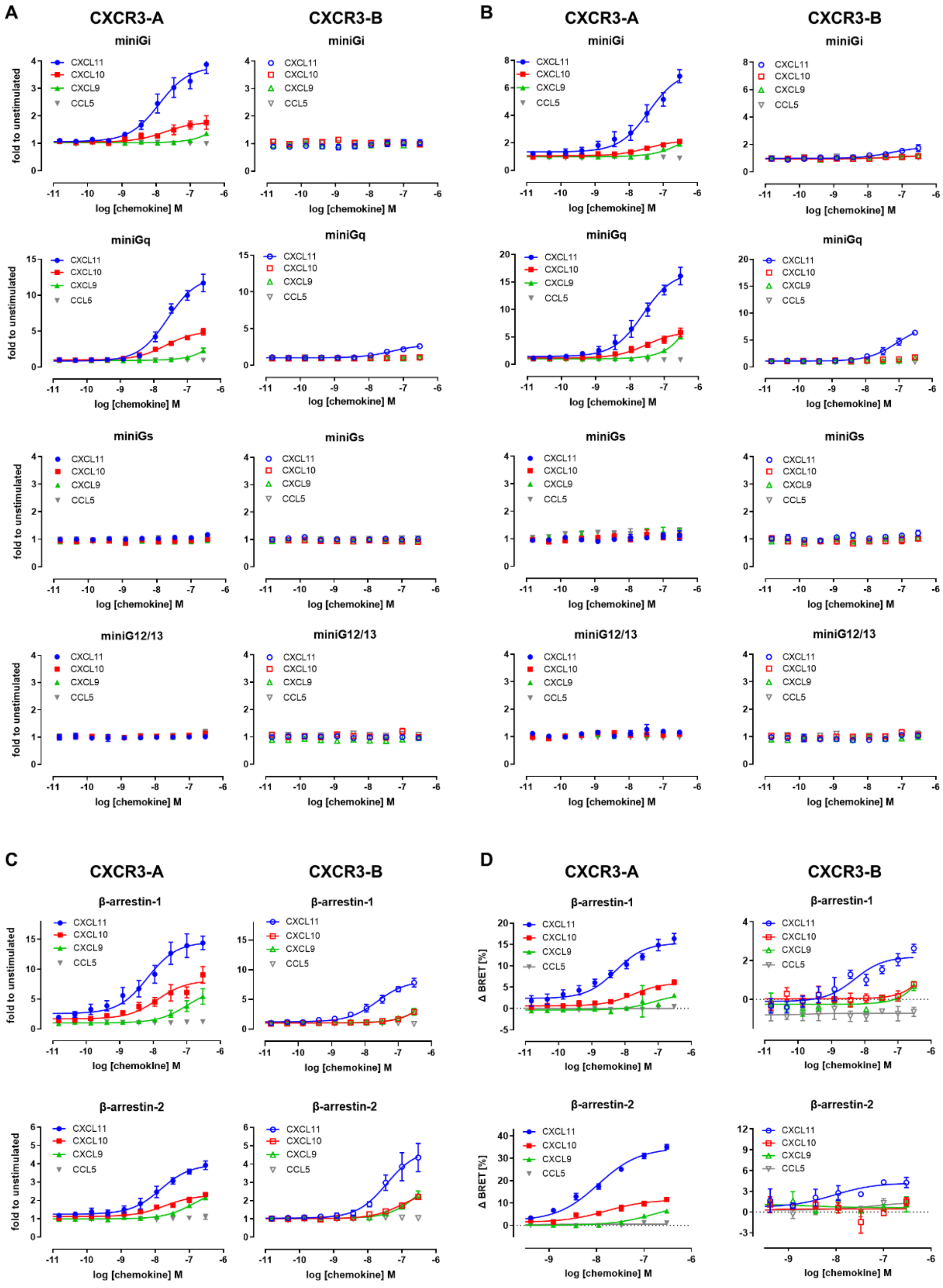

Supplementary figure 2. (**A–B**) Chemokine-induced recruitment of miniGi, miniGq, miniGs and miniG12/13 protein to CXCR3-A and CXCR3-B monitored by NanoBiT-based assay in HEK293T (**A**) or U-87 MG cells (**B**). (**C**) Chemokine-induced β-arrestin-1 and β-arrestin-2 recruitment to CXCR3-A and CXCR3-B after chemokine stimulation in U-87 MG cells using nanoluciferase complementation assay. (**D**) Chemokine-induced β-arrestin-1 and β-arrestin-2 recruitment to CXCR3-A or CXCR3-B monitored by NanoBRET assay in HEK293T cells. Data represent mean ± SEM of three independent experiments.

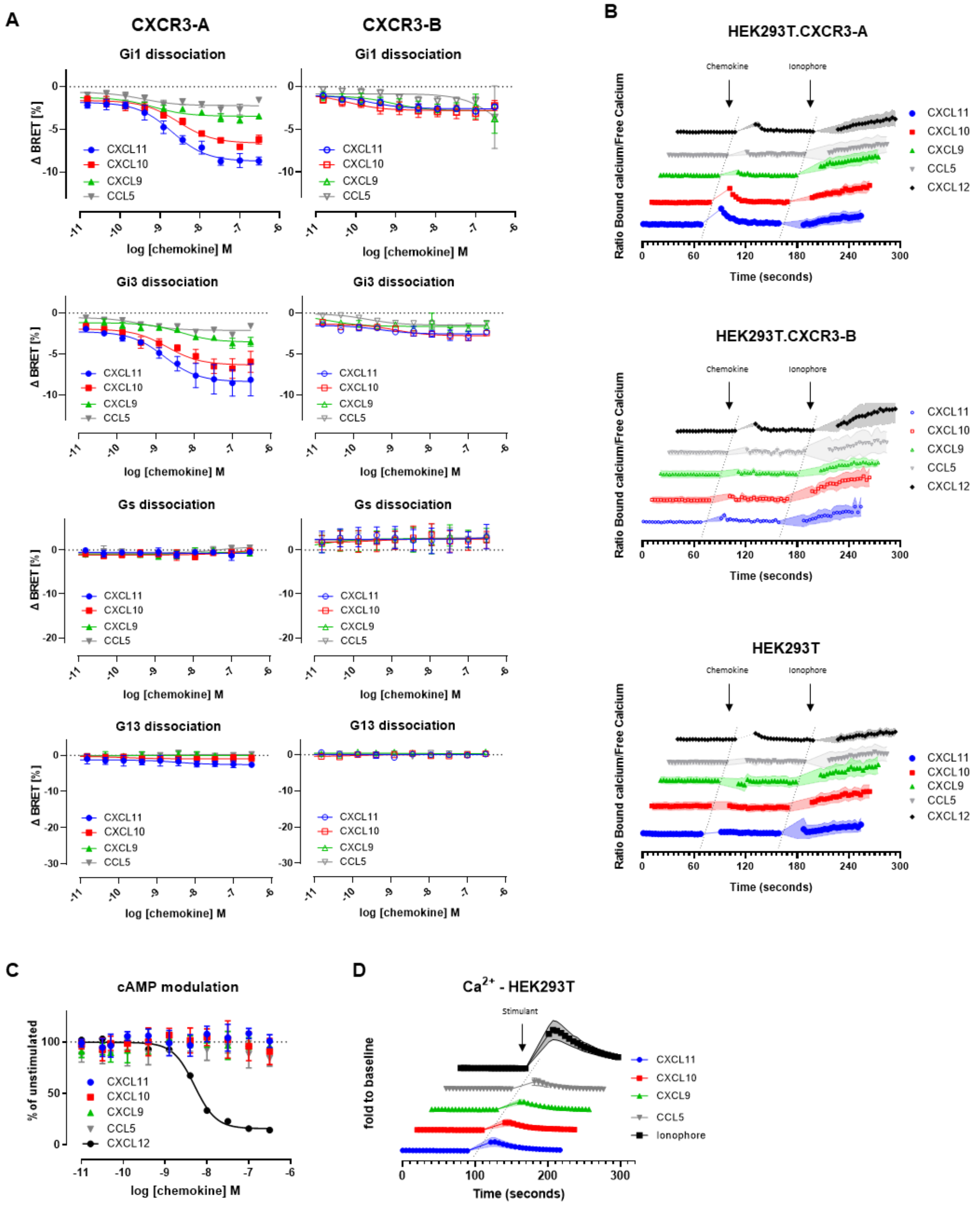

Supplementary figure 3. (**A**) Chemokine-induced heterotrimeric G protein dissociation profiles for G_{i1}, G_{i3}, G_s, G_{13} monitored in CXCR3-A- or CXCR3-B-expressing HEK293T cells by NanoBRET. (**B**) Mobilization of intracellular calcium in HEK293T cells stably expressing CXCR3-A or CXCR3-B or in naïve HEK293T cells in response to CXC or CC chemokines (100 nM) monitored with the ratiometric fluorescent indicator Indo-1 AM. Calcium ionophore A23187 (1 µM) was used as receptor-independent control. (**C–D**) Downstream G protein signaling in naïve HEK293T cells after treatment with CXCL12, CXCL11, CXCL10, CXCL9 or CCL5 by luminescence-based intracellular cAMP modulation (**C**) or NanoBiT-based intracellular calcium release (**D**). CXCL12 or Ionophore A23187 were used as positive control to confirm assay functionality. For all panels, data represent mean ± SEM of three independent experiments.

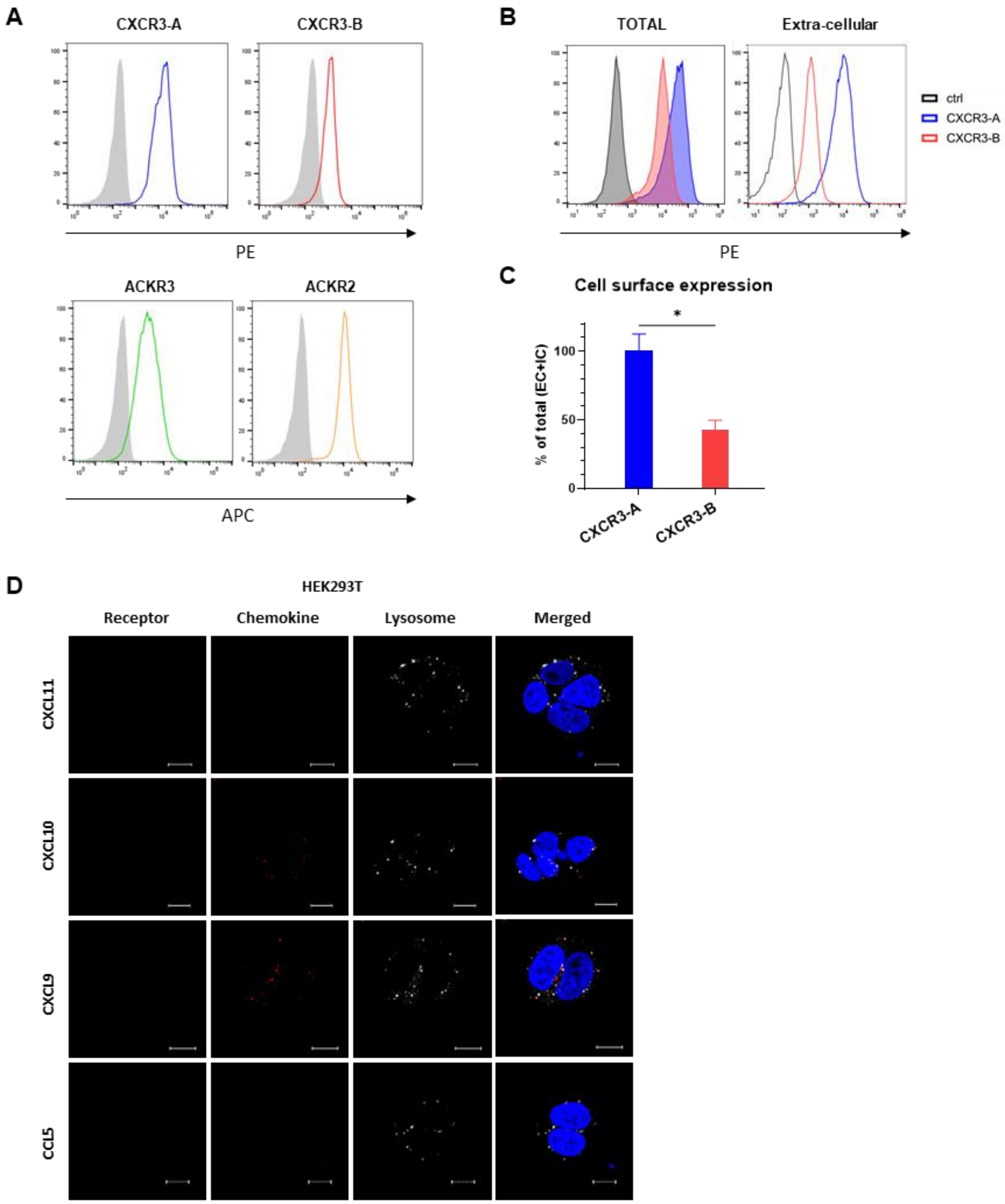

Supplementary figure 4. (**A**) Flow cytometry analysis of stable HEK293T-derived cell lines used in receptor recycling studies. Cell surface expression of CXCR3-A, CXCR3-B, ACKR3 or ACKR2, was evaluated with receptor-specific mAb (clones 1C6, 8F11-M16 and 196124, respectively) and the related isotype controls. **(B)** Flow cytometry comparison of total or extracellular expression of CXCR3-A and CXCR3-B HEK293T-derived stable cell lines evaluated with mAb 1C6 and related isotype control. (**C**) Cell surface expression of CXCR3-A and CXCR3-B quantified by HiBiT-mediated nanoluciferase complementation and expressed as percentage of total receptor expression (extracellular and intracellular). *$p < 0.05$ by Mann-Whitney two-tailed test. (**D**) Confocal microscopy images of naïve HEK293T cells after 2-hour incubation with CXCR3-related chemokines or CCL5 (100 nM). Alexa Fluor 647-labeled chemokines are represented in red, lysosomes stained with LysoTracker™ Red DND-99, in white and nuclei stained with Hoechst 33342, in blue. Scale bar: 10 µm. Pictures are representative of 12 acquired images from three independent experiments.

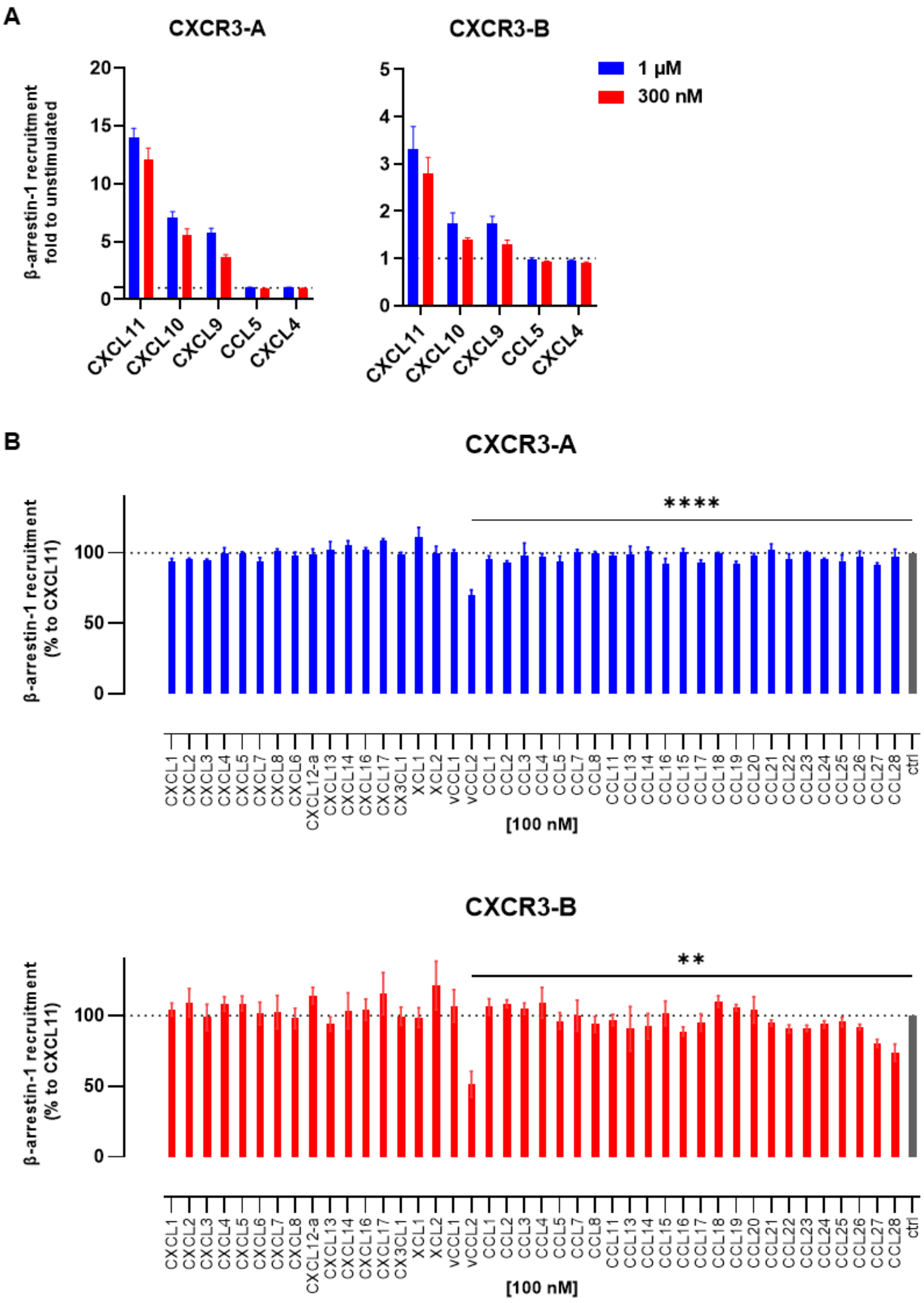

Supplementary figure 5. (**A**) β-arrestin-1 recruitment towards CXCR3-A and CXCR3-B in response to 1 µM or 300 nM of CXCL11, CXCL10, CXCL9, CCL5 and CXCL4 monitored by NanoBiT-based assay in HEK293T cells. (**B**) Antagonistic activity of all non-agonist chemokines (100 nM) towards CXCR3-A or CXCR3-B evaluated following addition of CXCL11 (20 nM) by NanoBiT-based β-arrestin-1 recruitment assay in HEK293T cells. **$p < 0.01$, ****$p < 0.0001$ by ordinary one-way ANOVA with Bonferroni multiple comparison test was used. For all panels, data represent mean ± SEM of three independent experiments.

Table 1 - CXCR3 variants activation by CXCL11, CXCL10 and CXCL9 (EC_{50} nM)

	CXCR3-A			CXCR3-B		
	**CXCL11**	**CXCL10**	**CXCL9**	**CXCL11**	**CXCL10**	**CXCL9**
miniGi recruitment	12.4 (7.2 – 21.6)	18.2 (3.9 – 74.0)	ND	NA	NA	NA
miniGq recruitment	25.9 (18.6 – 36.2)	22.2 (15.0 – 32.9)	ND	ND	NA	NA
β-arrestin-1 recruitment	15.8 (10.6 – 23.5)	17.8 (12.9 – 24.8)	ND	49.4 (36.9 – 66.6)	ND	ND
β-arrestin-2 recruitment	2.9 (1.8 – 4.8)	2.9 (0.8 – 10.2)	~ $1.6x10^4$	12.4 (7.3 – 21.2)	59.2 (23.5 – 159.7)	~ $1x10^5$
Gi2 dissociation	1.2 (0.4 – 3.9)	0.9 (0.3 – 2.7)	19.9 (5.9 – 54.4)	NA	NA	NA
Go1 dissociation	1.8 (0.8 – 4.2)	1.9 (1.1 – 3.1)	77.8 (34.1 – 201.5)	ND	NA	NA
Gq dissociation	0.4 (0.03 – 3.1)	2.3 (0.7 – 7.1)	~ 56	NA	NA	NA
cAMP	6. 3 (5.0 – 8.0)	14.1 (10.5 – 19.0)	21.9 (12.6 – 38.0)	NA	NA	NA

EC_{50} values are indicated in nanomolar (nM) with 95% confidence interval (CI).
ND: Not determinable since saturation was not reached.
NA: No activity or activity below 10% of positive control in the concentration range tested

Table 2 - N-terminally modified chemokines on CXCR3-A and CXCR3-B (EC_{50} nM and E_{max} %)

	CXCR3-A		CXCR3-B	
**Chemokines**	**EC_{50} nM**	**E_{max} %**	**EC_{50} nM**	**E_{max} %**
**CXCL11**	9.6 (5.6 – 16.3)	100	51.6 (40.7 - 65.3)	100.0
CXCL11-CD26	NA	6.2	NA	3.9
$CXCL11_{3-73}$	NA	7.5	NA	6.3
$CXCL11_{5-73}$	NA	0	NA	0.0
$CXCL11_{7-73}$	NA	5.8	NA	1.5
$CXCL11_{P2G}$	14.8 (9.8 – 22.5)	42.1	152.7 (107.8 – 226.4)	40.9
$CXCL11_{10Nloop}$	28.8 (22.8 – 36.4)	67.8	269.7 (168.2 – 504.6)	61.6
$CXCL11_{12Nloop}$	99.2 (67.7 – 150.4)	29.8	ND	10.6
**CXCL10**	10.7 (6.8 – 16.5)	41.7	ND	13.7
CXCL10-CD26	NA	1.2	NA	1.8
**CXCL9**	ND	28.1	ND	15.3
CXCL9-CD26	NA	9.5	NA	5.2

EC_{50} values are indicated in nanomolar (nM) with 95% confidence interval (CI).
Emax %: maximum signal measured at 300 nM expressed as % of the full agonist CXCL11.
ND: Not determinable since saturation was not reached.
NA: No activity or activity below 10% of positive control in the concentration range tested.

Table 3 - CXCR3-B N-terminal extension (EC_{50} nM and E_{max} %)

**miniGi**	**EC_{50} nM**	**E_{max} %**
CXCR3-A	18.2 (9.7 – 34.4)	100.0
CXCR3-B	NA	0.0
CXCR3-B -40	32.7 (11.4 – 97.2)	51.0
CXCR3-B -30	27.2 (6.5 – 128.0)	42.4
CXCR3-B -20	ND	11.6
CXCR3-B -10	ND	10.8
CXCR4	10.3 (4.6 – 23.2)	100.0
extCXCR4-B	ND	11.6
**β-arrestin-1**	**EC_{50} nM**	**E_{max} %**
CXCR3-A	6.7 (2.9 – 14.8)	100.0
CXCR3-B	23.9 (14.4 – 39.6)	75.9
CXCR4	7.1 (3.8 – 13.3)	100.0
extCXCR4-B	24.0 (12.6 – 46.1)	44.0

EC_{50} values are indicated in nanomolar (nM) with 95% confidence interval (CI).
Emax %: maximum signal measured at 300 nM expressed as % of the full agonist CXCL11.
ND: Not determinable since saturation was not reached.
NA: No activity or activity below 10% of positive control in the concentration range tested
CXCR3 artificial and natural isoforms were stimulated with CXCL11
CXCR4 artificial and natural isoforms were stimulated with CXCL12
